# Evolutionary Dynamics in the RNA Bacteriophage Qβ Depends on the Pattern of Change in Selective Pressures

**DOI:** 10.3390/pathogens8020080

**Published:** 2019-06-18

**Authors:** Pilar Somovilla, Susanna Manrubia, Ester Lázaro

**Affiliations:** 1 Centro de Astrobiología (CSIC-INTA), 28850 Torrejón de Ardoz, Madrid, Spain; psomovilla@cnb.csic.es; 2 Centro Nacional de Biotecnología (CSIC), 28049 Madrid, Spain; smanrubia@cnb.csic.es; 3 Grupo Interdisciplinar de Sistemas Complejos (GISC), Madrid, Spain

**Keywords:** RNA viruses, bacteriophage Qβ, adaptation, pattern of change, fitness dynamics, evolutionary pathways, genetic diversity

## Abstract

The rate of change in selective pressures is one of the main factors that determines the likelihood that populations can adapt to stress conditions. Generally, the reduction in the population size that accompanies abrupt environmental changes makes it difficult to generate and select adaptive mutations. However, in systems with high genetic diversity, as happens in RNA viruses, mutations with beneficial effects under new conditions can already be present in the population, facilitating adaptation. In this work, we have propagated an RNA bacteriophage (Qβ) at temperatures higher than the optimum, following different patterns of change. We have determined the fitness values and the consensus sequences of all lineages throughout the evolutionary process in order to establish correspondences between fitness variations and adaptive pathways. Our results show that populations subjected to a sudden temperature change gain fitness and fix mutations faster than those subjected to gradual changes, differing also in the particular selected mutations. The life-history of populations prior to the environmental change has great importance in the dynamics of adaptation. The conclusion is that in the bacteriophage Qβ, the standing genetic diversity together with the rate of temperature change determine both the rapidity of adaptation and the followed evolutionary pathways.

## 1. Introduction

Natural populations evolve in complex environments where multiple variables act interdependently, often experiencing non-uniform patterns of change. Establishing relationships among the values of environmental variables, changes in fitness, or the presence of specific mutations is, therefore, a difficult endeavor. In addition, there are many unknowns on the relevance of evolutionary history, chance, and natural selection in shaping the final evolutionary outcome. These complications can be partly dealt with in laboratory evolution experiments carried out with multiple replicas and under conditions imposed by the experimenter, facilitating the disentanglement of correspondences between genetic and phenotypic responses through time [1,2,3,4,5].

Many experimental evolution studies focus on adaptive dynamics following a sudden large change in an environmental condition that is kept constant [6,7,8]. However, natural environments rarely change in this simple way; often, environmental changes occur gradually, sometimes experiencing fluctuations that overlap the main trend. The rate of change of selective pressures may have profound consequences for the likelihood of adaptation or extinction of a population, for the followed mutational pathways, and for the maximum degree of adaptation achieved in the process [9,10,11,12,13,14,15,16].

Evolution in gradually changing environments has been modeled as a process of adaptation to a moving optimum [17,18,19,20]. The lower the rate of change, the smaller the drop in fitness the population experiences in each of the environments to which it is exposed. This means that the selective pressure under change has a low intensity at any point of the process and adaptation will occur through the selection of beneficial mutations that provide small effects on fitness [17,18,19,20]. Since these mutations are relatively abundant [21,22,23], there could be a diversity of accessible pathways for adaptation to gradual changes. In contrast to this, rapid environmental changes usually produce strong fitness drops that severely decrease the population size and, thus, the associated genetic diversity [24,25,26,27], making it difficult to find the scarce, large effect mutations that would make adaptation possible, unless those mutants already exist in the population, in which case the population bottleneck might help them to fix. The situation is even harsher when a particular combination of mutations is required for optimal adaptation. Due to the generally lower number of adaptive solutions to rapid changes, evolution would be more constrained under this condition than when changes take place at a slower rate [28]. Additionally, because epistatic interactions among mutations of small effect are weaker [29,30], it has also been suggested that gradual regimes would allow for populations to reach higher fitness values.

There are many experimental studies using a variety of biological systems, such as bacteria [12,31], unicellular algae [9], yeasts [14,16,32], or viruses [13,15,33], and selective pressures, such as antibiotics [12,31], heavy metals [14,16], new hosts [13,15], salt stress [32], or changes in temperature [33], that have explored the influence of the rate of environmental change on adaptation. In good agreement with the predictions of the models, in some studies the rate of population extinction was higher under more rapid environmental changes [12,31,32,33] due to the strong decline in population sizes and also, in some cases, because the adapted genotypes were only accessible upon a previous sequence of environments. This kind of historical contingency can occur when certain mutations that permit survival at high intensity of the selective pressure are deleterious in the absence of other mutations that can only be selected at a lower intensity of the selective pressure [12,34]. There are also examples showing that populations evolving under gradual changes reached higher fitness values [9,13,25] and/or displayed more genetic diversity among replicate lineages than those evolving under more abrupt changes [9,12]. In contrast to this, the results obtained in other studies manifested some discrepancy with the prediction of the models, showing that phenotypes obtained under rapid environmental change were fitter [31,32,35] or contained a greater diversity of mutations than those evolving at a slower pace [13]. Finally, other studies did not find differences either in the fitness or in the genetic diversity contained in populations that could be attributed to the rate of environmental change [14,16,36]. Altogether, those results suggest that the differences in evolutionary outcomes due to the rate of change in the selective pressures depend on both the biological system—with its particular mutation and replication rates, relevance of epistatic interactions, and underlying distribution of mutational effects—and on the assayed environmental condition.

In this work, we studied how a temperature increase of fixed magnitude but variable rate influenced evolutionary outcomes in an RNA bacteriophage. Specifically, we used as a model the bacteriophage Qβ, of the family *Leviviridae*, which infects *Escherichia coli* strains expressing the F pilus that acts as the virus receptor [37]. Bacteriophage Qβ has an RNA genome of positive polarity (such as poliovirus, foot-and-mouth disease virus, or the coronavirus causing the severe acute respiratory syndrome) of 4217 nucleotides that encodes four proteins: the A2 protein for bacterial lysis and entry, the coat protein, the A1 protein, which is present in low amounts in the capsid and expressed through incorrect reading of the stop codon of the coat protein, and the replicase that copies the RNA genome [37]. As in other RNA viruses, replication in Qβ proceeds at a high error rate [38], giving rise to highly heterogeneous populations that adapt fast. The optimal temperature for replication of this virus is 37 °C (the host temperature).

Much of the difficulty in treating diseases caused by viruses, especially RNA viruses, stems from their high evolutionary potential [39,40,41]. Infection of new hosts, adaptation to antivirals, or the emergence of variants that cannot be neutralized by antibodies are only a few examples of the great adaptability of RNA viruses to changes in environmental variables. Therefore, to find effective control therapies, it is necessary to improve our knowledge of their evolutionary possibilities, for which RNA bacteriophages can be an appropriate, and easy to handle, model. In this study, the temperature for Qβ replication was increased from 37 °C to 43 °C either suddenly or following two patterns of gradual change. Due to the importance of the standing genetic diversity prior to adaptation [42], we also compared the evolutionary pathways followed by the virus when populations that differed in their degree of diversification from a clonal origin were propagated at 43 °C upon a sudden exposure to this temperature.

There are previous studies concerning the adaptation of Qβ to replicate at a high temperature. In some of them [43,44,45], the used virus variant, which could be directly propagated at 43 °C, was obtained upon expression of an infectious clone (pBRT7Qβ) containing a cDNA of the virus genome cloned in pBR322 [46,47]. In another work [34] carried out with a different virus variant (obtained upon expression of the infectious clone pACYCQβ [48]), the temperature had to be gradually increased (in three steps) from 37 °C to 43.6 °C degrees to avoid virus extinction. Alignment of the virus genomes cloned in pACYCQβ and pBRT7Qβ showed the presence of two silent substitutions in the latter (C1257U and C2249U). These substitutions, together with G4A, U192C, and C2201U (also silent), were a necessary requirement for the virus obtained upon expression of pACYCQβ to adapt to a high temperature [34]. Since a variant that could replicate at 43 °C was needed to analyze the adaptive process of Qβ as a function of the rate of temperature change, the virus populations used in this work were all generated from the infectious clone pBRT7Qβ.

The main objectives of this work were: (1) to compare the dynamics of fitness gains at 43 °C and the fitness values that were reached at the end of the transfer series, and evaluate their dependence on the rate of temperature increase; (2) to analyze the similarities of adaptive pathways among evolutionary lineages subjected to the same or a different pattern of change; (3) to identify mutations specific to a particular temperature or range of temperatures; and (4) to analyze the influence of the standing genetic diversity on the dynamics of adaptation and on the followed evolutionary pathways.

## 2. Results

### 2.1. Evolution of Bacteriophage Qβ Under Different Patterns of Temperature Increase

**Figure 1:**
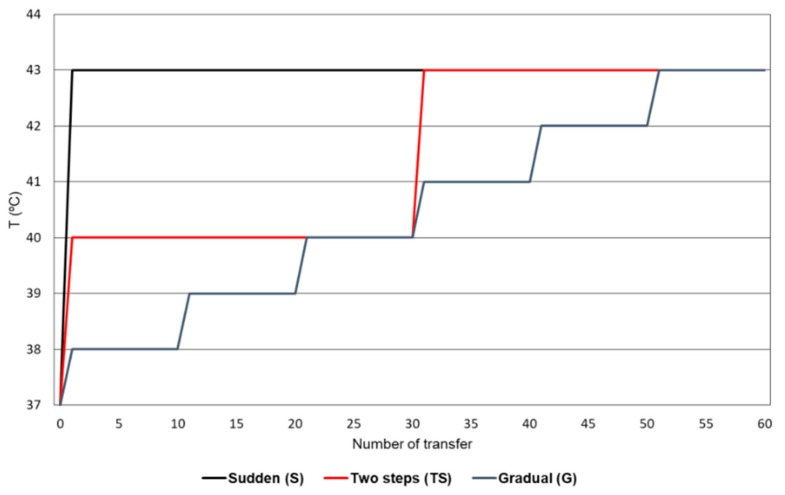
Patterns of temperature increase followed by the Qβ evolutionary lineages. The sudden pattern (S) consisted of 60 transfers carried out at 43 °C. In the two-step pattern (TS), the virus was propagated for 30 transfers at 40 °C followed by 30 additional transfers at 43 °C. The gradual pattern (G) involved an increase of 1 °C every 10 transfers.

Populations Qβ-t2 and Qβ-t25, obtained upon propagation of a clonal virus for two and 25 transfers at 37 °C, respectively (see Materials and Methods), were the origin for further evolution at an increased temperature, following three patterns of change (also called treatments from now on), that differed in the rate at which the maximum value (43 °C) was reached (Figure 1). The sudden pattern (S) entailed an abrupt exposure to 43 °C, after which the temperature was kept constant. The remaining two patterns involved a progressive temperature change: a gradual increase of 1 °C every 10 transfers (pattern G) or a two-step increase consisting of 30 transfers at 40 °C and 30 transfers at 43 °C (pattern TS). Population Qβ-t25 was propagated through the three patterns of change, giving rise to the evolutionary lineages S-t25, TS-t25, and G-t25. Population Qβ-t2 was only subjected to the sudden treatment, giving rise to the evolutionary lineages S-t2. All propagations were carried out in triplicate for a total of 60 transfers at a higher-than-optimal temperature (see Materials and Methods). Note that the number of transfers experienced at 43 °C was different for different populations: 60, 30, and 10 transfers in populations evolved through patterns S, TS, and G, respectively.

**Figure 2:**
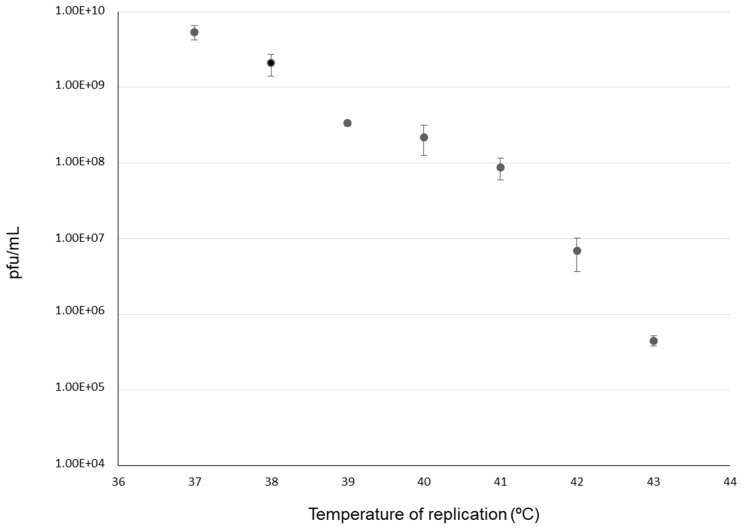
Sensitivity of bacteriophage Qβ to increased temperature. Virus replication was assayed by infecting 1 mL *Escherichia coli* cultures (containing ~10^8^ bacteria) with 10^4^ plaque forming units (pfu) from the virus population Qβ-t2. After incubation for 2 h at the temperatures indicated in the figure, virus supernatants were collected as described in Materials and Methods, and titrated to estimate the virus yield, which was expressed as the total number of pfu per mL of culture. Each determination corresponds to the average of two replicas and the error bars represent the standard deviation.

The intensity of the selective pressure caused by the increase of temperature was analyzed in an assay in which we determined the virus yield for population Qβ-t2 at different values of this parameter (Figure 2). As expected, the highest titers were obtained at 37 °C. Temperatures above this value decreased the virus yield in an almost exponential way.

### 2.2. Dynamics of Adaptation to 43 °C

**Figure 3:**
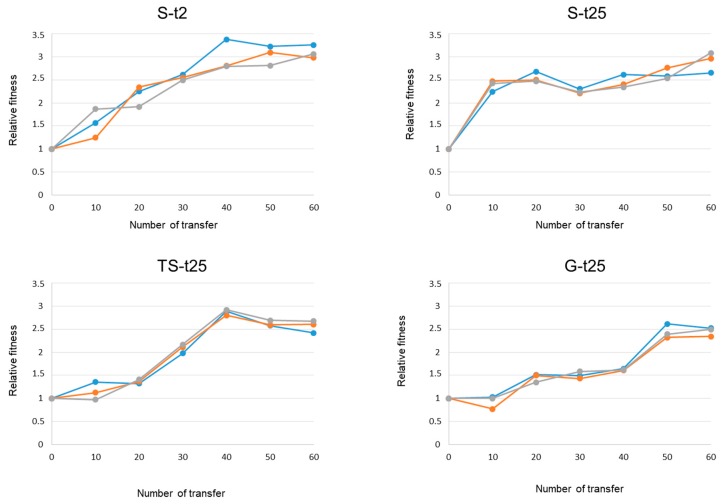
Fitness dynamics at 43 °C of the Qβ evolutionary lineages propagated through the different patterns of temperature increase. In each treatment, the blue line represents the evolutionary lineage 1, the orange line the lineage 2, and the grey line the lineage 3. Relative fitness values were calculated as described in Materials and Methods. Each point represents the average of two replicas. The standard deviation was always lower than 10%.

To analyze the dynamics of adaptation of Qβ to increased temperature, we determined the fitness values of the virus at 43 °C in all evolutionary lineages every 10 transfers (see Materials and Methods). Values were referred to that of the corresponding ancestor population (4.7 ± 0.1 for Qβ-t2 and 4.8 ± 0.7 for Qβ-t25). The similarity between fitness values of populations Qβ-t2 and Qβ-t25 indicates that the previous evolution at 37 °C did not significantly increase fitness at 43 °C. In contrast to this, results obtained with the lineages evolved at a higher-than-optimal temperature showed that virus propagation at temperatures above 38 °C always produced fitness increases at 43 °C (Figure 3). Both the rate of temperature increase and the previous history of the ancestor population influenced fitness dynamics, as observed in the different adaptive trajectories that were followed (Figure 3). Fitness increased much faster in populations suddenly exposed to 43 °C, particularly in lineages S-t25, in which most of the increase took place during the first 10 transfers. Lineages S-t2 increased their fitness more slowly than lineages S-t25, and almost linearly until transfer number 40. In lineages evolving through the progressive treatments, the largest fitness increases took place between transfers 20 and 40 in lineages TS-t25 (the last 10 transfers at 40 °C and the first 10 transfers at 43 °C), and between transfers 10 and 20 (39 °C) and transfers 40 and 50 (42 °C) in lineages G-t25.

To make quantitative comparisons among the different evolutionary lineages, we determined the fitness values that were reached at the end point of evolution (transfer 60), the fitness differences that were attained during the first 10 transfers at 43 °C, and the number of transfers that was necessary to duplicate the fitness of the ancestor population (that is, to reach a relative fitness value of 2). The values for all of these parameters are shown in Table 1.

Populations that were exposed directly to 43 °C (lineages S-t2 and S-t25) reached statistically similar fitness values at transfer number 60 (*p* > 0.1; Student’s *t*-test). The same happened in populations that reached 43 °C progressively (*p* > 0.1 for the comparison of lineages G-25 versus TS-t25; Student’s *t*-test). On the other hand, comparison of fitness values between populations that evolved through different patterns of temperature increase showed that sudden exposure to 43 °C allowed the virus to reach slightly higher, but significantly different, fitness values than propagation through the TS or G treatments (*p* < 0.05 for any comparison of the set of lineages S-t25 or S-t2 with either TS-t25 or G-t25; Student’s *t*-test).

The variation in the fitness values during the first 10 transfers carried out at 43 °C (this is from transfer 1 to 10 in lineages S-t2 and S-t25, from transfer 30 to 40 in lineages TS-t25, and from transfer 50 to 60 in lineages G-t25) showed marked differences depending on both the ancestor population and the pattern of temperature increase (Table 1). In populations with the same ancestor (lineages S-t25, TS-25, and G-t25), the lower the fitness value before the first exposure to 43 °C the larger the fitness gain after 10 transfers at this temperature. In populations that evolved through the sudden treatment and differed in their ancestors, fitness gains were much higher in lineages St-25 than in S-t2 (*p* < 0.05, Student’s *t*-test).

There were also important differences in the number of transfers required to duplicate the initial fitness value (Table 1). In lineages with the same origin (S-t25, TS-t25, and G-t25), the slower the temperature increase, the greater the number of transfers needed to double the initial fitness (*p* < 0.001 for all possible pair-wise comparisons between the different sets of lineages, Student’s *t*-test). The importance of the ancestor population was manifested in the fact that populations S-t2 needed an average of 18.2 transfers to reach a fitness value of 2, whereas St-25 needed only an average of 7.3 (*p* < 0.01, Student’s *t*-test).

### 2.3. Analysis of the Consensus Sequences of Final Qβ Populations

**Figure 4:**
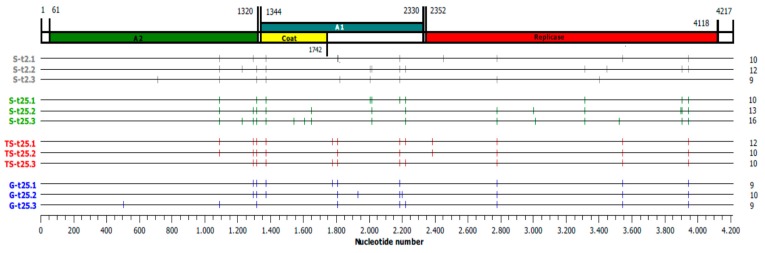
Mutated positions present at transfer number 60 in the consensus sequences of the Qβ evolutionary lineages propagated through the different patterns of temperature increase. All mutations (fixed and polymorphic) relative to the sequence of the Qβ cDNA cloned in pBRT7Qβ (see Materials and Methods) are represented as vertical lines on the horizontal lines corresponding to the Qβ genome. The total number of mutations in each genome is shown on the right part of the figure.

We determined the consensus sequences of the two ancestor populations, Qβ-t2 and Qβ-t25, and of the 12 evolutionary lineages obtained at transfer number 60. Qβ-t2 did not show any mutation relative to the Qβ genome cloned in pBRT7Qβ, and Qβ-t25 presented only two polymorphic substitutions: A2187C (S281R in the A1 protein) and C3065U (synonymous). Both populations presented a uracil at positions 1257 and 2249, which is a characteristic of pBRT7Qβ that facilitates adaptation to a high temperature (see Introduction). In the evolved populations, we found a total of 130 mutations (including fixed and polymorphic), which were located in 35 nucleotide positions (Figure 4 and Table 2). There were no statistically significant differences in the number of mutations per genome (Figure 4) that could be attributed to the pattern of temperature increase (*p* > 0.05 for all possible pair-wise comparisons between different sets of lineages, Student’s *t*-test). Mutation C3065U, which was present in the ancestor Qβ-t25, was lost in all lineages founded by this population. However, since the mutation was polymorphic in Qβ-t25, we cannot know whether the loss was due to a reversion or to negative selection, though we believe the latter is more plausible. Substitution A2187C, also in Qβ-t25, presented a non-uniform behavior. It was lost in some lineages, whereas in others was fixed or remained polymorphic.

From the set of 130 mutations, 105 (located in 23 nucleotide positions) were non synonymous, which is a clear excess over the number of synonymous mutations (Table 2). Sixteen substitutions were exclusive of only one evolutionary lineage, whereas others were represented in at least half of the lineages (A1088G, U1295(C/G), G1312A, G1371A, C1806U, U2776C, C3545U, and G3945A). All but one (C3545U) of these highly represented substitutions were non synonymous and distributed similarly across all treatments (again with the exception of C3545U, which was more frequent in the progressive treatments).

**Figure 5:**
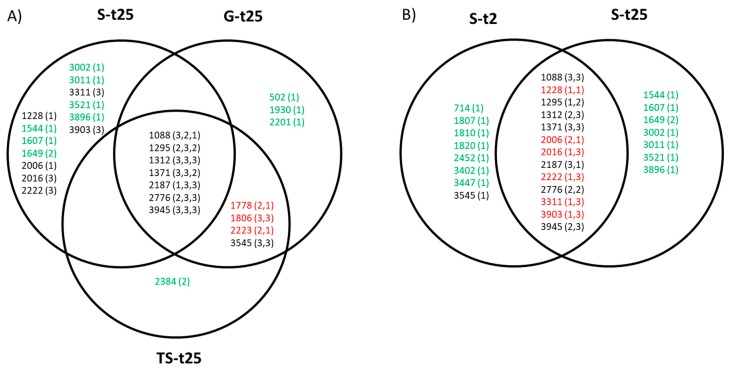
Representation of mutated positions at transfer number 60 in the sets of lineages propagated through different patterns of temperature increase. (**A**) Lineages with the same origin and subjected to different treatments. (**B**) Lineages subjected to the sudden treatment and differing in their ancestor population. Each circle encloses the nucleotide positions that are mutated at least in one evolutionary lineage of each set. The numbers between brackets indicate the number of times that a particular position is mutated per set of lineages evolving under the same condition. The order of the numbers for positions common to several sets is: (S-t25, TS-t25, G-t25) or (TS-t25, G-t25) in part A), and (S-t2, S-t25) in part B). Mutated positions common to S-t2 and S-t25, and non-mutated in the progressive treatments, and conversely are shown in red. Mutated positions exclusive of each set of replicate lineages are shown in green.

The distribution of mutated positions common to and exclusive of the different treatments is represented in Figure 5. When comparing the lineages with the same origin and evolving through different patterns of temperature increase (Figure 5A), we can observe that there are some substitutions (A1088G, U1295(C/G), G1312A, G1371A, A2187C, U2776C, and G3945A) that were present in at least one lineage of each of the three sets S-t25, TS-t25, and G-t25. The same substitutions, together with six additional ones, were also represented in at least one lineage of the two sets St-2 and St-25 (Figure 5B). There were also some substitutions common to S-t2 and S-t25 and absent from the progressive treatments, and conversely (in red in Figure 5). The number of different substitutions exclusive of each set of replicate lineages was 7 for S-t25 and S-t2, 1 for TS-t25, and 3 for G-t25 (in green in Figure 5). Most of them were present in a single lineage.

### 2.4. Evolutionary Convergence among Lineages and Treatments

The nucleotide diversity among replicate lineages was evaluated as the number of pair-wise differences between genomes divided by the number of analyzed nucleotides. The results showed that lineages S-t2 had higher nucleotide diversity than lineages St-25 (3.2 × 10^−3^ vs. 2.1 × 10^−3^). Evolution through any of the progressive treatments led to lower nucleotide diversity (1.4 × 10^−3^ for lineages G-t25 and 7 × 10^−4^ for lineages TS-t25) than evolution from the same ancestor and through the sudden treatment (lineages S-t25). The conclusion is that both the characteristics of the ancestor population and the pattern of temperature increase influence the nucleotide diversity among replicate lineages.

Convergence between different sets of replicate lineages was evaluated by counting the number of mutations that were present in at least one lineage of each set, and dividing it by the total number of different mutations in the two sets. Convergence between lineages evolved through a similar treatment had a value of 0.46 for lineages S-t2 versus S-t25 (sudden treatment), and of 0.73 for TS-t25 versus G-t25 (progressive treatments). That value decreased when populations evolved under any of the progressive patterns were compared to those subjected to the sudden treatment (0.26 for lineages G-t25 vs. S-t25 and 0.28 for lineages TS-t25 vs. S-t25). Altogether, the results show that the pattern of temperature change is more important than the ancestor population to determine the mutations that can be selected throughout the adaptive process and that contribute to define the degree of convergence between different treatments.

### 2.5. Evolution of the Consensus Sequences through Adaptation

With the aim of analyzing the dynamics of adaptation at the genetic level, we determined the consensus sequence of all evolutionary lineages every 10 transfers (Supplementary Figure S1). Representation of the total number of mutations (fixed and polymorphic) as a function of the number of transfers (Supplementary Figure S2) allowed us to investigate whether the pace of mutation acquisition mimicked the dynamics of adaptation shown in Figure 3. The results showed that, during the first 10 transfers, lineages S-t25 acquired mutations much more rapidly than the rest (*p* < 0.001 for all comparisons between the set of lineages S-t25 with any other set; Student’s *t*-test), which agrees with their faster adaptation at early stages. At transfer number 20, the differences were lower but still statistically significant (*p* < 0.05 for all comparisons between the set of lineages S-t25 with any other set; Student’s *t*-test). From transfer 40 onwards, there were no significant differences in the number of mutations between any set of evolutionary lineages.

We also determined the mutations that first fixed in each evolutionary lineage and the number of transfers at which they fixed (Table 3). In populations TS-t25 and G-t25, the first mutation fixed was G1312A (in all evolutionary lineages but G-t25.2, where A1930G was the first mutation fixed), and only in one case (TS-t25.2) two mutations fixed simultaneously (A1930G, G1312A). All these substitutions fixed at transfer number 20, with the exception of G1312A in TS-t25.3 that fixed at transfer number 30. Populations evolved through the sudden treatment yielded different quantitative results. The first event of fixation of mutations involved a wider set of mutations and, in the case of lineages S-t25, it also took place earlier (at transfer number 10). Remarkably, substitutions A1930G and G1312A, which were the first to be fixed in the progressive treatments, were not found amongst the first fixed in the sudden treatment, with the only exception of G1312A in S-t2.3. Substitution A1930G was not present at any transfer in any of the lineages evolved constantly at 43 °C, and was lost or became less conspicuous in all lineages evolving through the progressive treatments. Remarkably, most of the substitutions among those first fixed in lineages S-t25 (C1649U, U2016C, A2222C, U2460C, and U3311G) were absent in the lineages TS-t25 and G-25, whereas A1088G was only present as a polymorphism at late transfers.

There were also some mutations (between four and six per treatment) that were present at some point of the transfer series, and disappeared or decreased their representation at later transfers. The most noticeable example was the aforementioned substitution A1930G. In some cases, lost mutations were fixed in other lineages and, in other cases, substitutions that were becoming less abundant increased their frequency anew at later transfers.

### 2.6. Fixation of Mutations and Temperature of Evolution

Populations subjected to the progressive temperature increases evolved through a series of different environments, each of them characterized by a temperature value. The analysis of the mutations selected in each of these environments may shed some light on the fitness effects of mutations at different temperatures. We determined which mutations were detected for the first time in the range between 38 °C and 40 °C or at temperatures above 40 °C in the lineages TS-t25 and G-t25. Most mutations appeared for the first time in the range of 38–40 °C (U1295C/G, G1371A, G1312A, C1806U, A1930G, A2187C, C2201U, U2776C, C3545U, and G3945A) (Supplementary Figure S1). Some of these substitutions were also present in most of the lineages evolving constantly at 43 °C (G1312A, U1295C/G, G1371A), suggesting that they are beneficial at all assayed temperatures. In contrast to this, C1806U, A1930G, and C2201U were absent from all the lineages evolved at a constant 43 °C, while A2187C and C3545U were present only in one. Whereas A1930G exhibited a significant propensity to be lost at later transfers, suggesting a negative effect at higher temperatures, C1806U remained in all lineages and C2201U in one (Supplementary Figure S1). Substitutions A1088G, A1778G, G2223A, and C2384T were first detected at temperatures above 40 °C (Supplementary Figure S1). All except A1778G were also present in the lineages evolving at a constant 43 °C.

## 3. Discussion

The results obtained in this work confirm the conclusions of previous studies showing that both the rate of change in the selective pressures and the characteristics of populations before exposure to new conditions have a great influence on the dynamics of adaptation, the achieved fitness values, the mutational pathways followed, and the repeatability of evolution [9,10,11,12,13,14,15,16]. The properties of our experimental model—an RNA phage with high mutation rates, fast replication, and large population sizes [38,49]—and of the assayed selective pressure—an environmental condition that affects most biological processes—may determine some of the particularities of our findings.

Previous studies carried out with Qβ showed a discrepancy in the capacity of this virus to withstand high temperatures. Whereas the virus obtained upon expression of the infectious clone pACYCQβ [48] was unable to produce an infectious progeny at 43.6 °C [34], the virus obtained upon expression of pBRT7Qβ [46,47] could replicate at 43 °C, although rendering much lower titers than at 37 °C [44]. The ability of pBRT7Qβ to generate viruses able to replicate at 43 °C was probably due to the presence of two silent substitutions (C1257U and C2249U) that, together with G4A, U192C, and C2201U (also silent), allowed for adaptation to a high temperature of the virus obtained upon expression of pACYCQβ [34]. Optimal adaptation also needed the fixation of substitutions A1088G, A2748C, and U2776C, whose beneficial effects were dependent on the presence of the aforementioned set of five silent substitutions. As we have shown in this work, Qβ obtained upon expression of pBRT7Qβ could efficiently adapt to replication at 43 °C both when the temperature increased sharply and when it did in a more gradual manner. Thus, the presence of only C1257U and C2249U sufficed to permit virus replication at 43 °C, keeping population sizes compatible with optimal adaptation. Substitutions A1088G, U2776C, and C2201U were also detected in the evolutionary lineages analyzed in this work, all with origin in pBRT7Qβ. Altogether, the results obtained with both Qβ variants show the relevance of the genetic background to determine the fitness effects of mutations [3,4,50,51,52,53], in this case of those with beneficial effects at a high temperature. Since some of the mutations that facilitate adaptation of Qβ to increased temperature were already present in the virus prior to high-temperature exposure, it would be expected that they could exert similar effects under other unrelated selective pressures.

The comparison of the dynamics of fitness gains in populations with the same origin but differing in the rate of temperature increase (S-t25, TS-t25, and G-t25) showed that virus propagation at temperatures above 38 °C produced fitness increases at 43 °C, suggesting that at least some of the mutations selected when the selective pressure was mild were also beneficial at a higher intensity. However, neither the fitness gains reached when the evolution experiment was stopped nor the fitness trajectories along the transfer series were similar. The lower the rate of temperature increase, the larger the number of transfers needed to duplicate the fitness of the ancestor, which agrees with the selection of mutations of smaller effect at lower intensities of the selective pressure, as predicted by theoretical models [17,18,19,20]. By transfer number 60, sudden exposure to 43 °C also led to fitness values higher than those reached under progressive temperature changes, as observed elsewhere [31,32,35]. Differences in the fitness dynamics could be well-explained if, as mentioned above, the mutations selected in lineages subjected to different treatments had different effects on fitness and/or they accumulated at a different rate.

In good agreement with rapid increases in fitness at early transfers, the first event of fixation in lineages St-25 involved more mutations and took place earlier than in lineages evolving under more progressive change. The fast selection of these mutations in lineages S-t25 suggests that their ancestor population probably contained some genomes that either had beneficial effects at 43 °C or could facilitate the acquisition of adaptive mutations at this temperature. The first fixed mutations were not the same in the different treatments, which could indicate that the magnitude of their effects, or even the sign of some of them, depended on the intensity of the selective pressure, something that has also been demonstrated in previous studies [12,34]. The lower fitness values reached at transfer 60 by populations evolved through the progressive treatments could also be explained if the mutations acquired during transfers carried out at lower temperatures entailed fitness costs at higher temperatures or imposed restrictions on the mutations acquired at later transfers. In line with this argument, some of the first fixed mutations in lineages S-t25, probably responsible for their large fitness increases at early transfers, were not present in the lineages evolved at a progressively increased temperature. The clearest example of a mutation with a probable fitness cost at a high temperature was A1930G, which was only present in lineages G-t25 and TS-t25 at early transfers—to disappear later as the temperature rose—and was always absent in lineages S-t25.

Populations evolving through the different treatments had different life stories, and therefore different fitness values before the first exposure to 43 °C. There was a negative linear relationship between those values and the fitness gains upon the subsequent 10 transfers at 43 °C, confirming the general observation in micro-organisms that fitter populations adapt more slowly than less fit populations [54,55,56,57]. This decrease in the rate of adaptation indicates that either better-adapted populations have already used most of the beneficial mutations available or that beneficial mutations have weaker effects on fitter genotypes. The latter is a form of epistasis known as diminishing returns epistasis [58,59], identified in previous studies, that seems to represent a generic negative coupling among beneficial mutations. Diminishing returns epistasis could explain why some of the mutations responsible for the largest fitness increases in poorly adapted populations (population Qβ-t25 before propagation through the sudden treatment) were not selected when better-adapted populations were also exposed to 43 °C (lineages TS-25 at transfer number 30 and lineages G-t25 at transfer number 50).

Convergence among replicate lineages was higher for populations evolved through progressive temperature increases than for populations constantly evolved at 43 °C. Although sudden exposure to a harsh selective pressure might have caused the selection of the genomes with the most beneficial mutations that were already present in the standing genetic diversity of the ancestor population, the large number of transfers experienced at 43 °C by lineages evolving under constant conditions might have caused their divergence due to the acquisition of new mutations of small effect. Convergence between treatments was higher when lineages TS-t25 were compared with lineages G-t25 than when any of them was compared with lineages S-t25, highlighting the importance of the pattern of change in the followed evolutionary pathway.

The comparison of the dynamics of adaptation to 43 °C in lineages S-t2 and S-t25, both propagated through the sudden treatment but differing in their ancestor population, shows great differences in the rate of adaptation, which was much faster for lineages S-t25. Due to differences in their life histories, both ancestors Qβ-t2 and Qβ-t25 probably contain different genetic diversity, arguably lower in population Qβ-t2 than in Qβ-t25. The rapidity of adaptation in lineages S-t25 suggests the presence in their ancestor populations of some genomes containing mutations that facilitate adaptation to 43 °C. These genomes should be present in low frequencies, since population Qβ-t25 does not have higher fitness than Qβ-t2 at 43 °C. In contrast to this, adaptive mutations were probably absent from population Qβ-t2 and had to be generated de novo, which causes the lower rate of adaptation in lineages S-t2. This fact can also underlie the higher nucleotide diversity among replicate lineages and the larger number of transfers that is required for the fixation of the first set of mutations in lineages S-t2 as compared to lineages S-t25.

Although lineages S-t25 adapted faster than lineages S-t2, they reached similar fitness values at the end point of evolution. There was also high mutational convergence between both sets of lineages, higher than between any of them and Gt-25 or TS-t25, indicating that the pattern of change in temperature is more important than the ancestor population to determine which mutations will be selected for optimal adaptation. The main differences in the consensus sequences of lineages St-25 and S-t2 at transfer number 60 were due to mutations represented in only one evolutionary lineage. Those mutations could not have selective value and may have been fixed through hitchhiking. Most represented mutations identified in this study were also detected in previous studies in which Qβ adapted to a high temperature [34,43,44,45]. This agreement notwithstanding, most of the mutations (12 out of 16) that were present in only one evolutionary lineage had not been previously detected during adaptation of Qβ to a high temperature.

There are multiple fitness traits that can be associated with increased performance of Qβ to high temperatures, among them the extracellular stability of the virus particles, the adsorption rate to the host, the time span that is required to produce an intracellular progeny, and the liberation of the new-formed viruses to the external medium. Kashiwagi et al. [60] prepared several site-directed mutants containing different sets of mutations among those selected at a high temperature, and determined some of the parameters that characterize the Qβ infective cycle. All the combinations of assayed mutations increased the phage adsorption rate and decreased the latent period at 43.6 °C, which agrees with adaptation to this temperature. However, surprisingly, the same site-directed mutants displayed decreased thermal stability at 43.8 °C, which was attributed to the presence of substitution A1088G (D342G) in the A2 protein. Nucleotide D342 is placed near the surface of the capsid and has been suggested to interact with the coat protein. Thus, mutations at this position can alter the stability of the capsid [61]. Further experiments showed that stability could be restored, at least partially, through silent mutations that affect the structure of the RNA. Other substitutions that may have an effect on virus stability are C1649U (synonymous, in the coat protein), A1930G (Q195R in the A1 protein), U2016C (F224L in the A1 protein), and G3945A (G531S in the replicase). All of them were identified in an experiment designed to select an increased virus multiplication rate at 37 °C, which was associated with decreased stability at the same temperature [62]. Altogether, these results illustrate how the optimization of some fitness traits entails a cost in others, requiring the presence of additional mutations to generate an optimized genotype. Currently, the crystal structure of all the Qβ proteins has been determined [61,63,64,65,66], opening the door to computational studies able to assess whether a particular mutation (or combination of mutations) influences the stability of the protein. Similar studies could be carried out with the genomic RNA.

The elucidation of the molecular mechanisms by which each of the mutations identified in this work exert its effects is a topic of interest that deserves further attention. The analysis of the mutational dynamics at the molecular level would need to track the changes in the frequency of particular substitutions through time by means of high-throughput sequencing techniques. Determination of the consensus sequences of sets of biological clones at different points of the process would be very useful to determine which associations of mutations are favored (such as U2016C, U3311G, and C3903U, which appear always together in the same lineages, suggesting that their positive effect could be dependent on their joint presence), and to determine the intra-population dynamics of adaptation. These studies are currently under progress and will help to better elucidate the relationships between the pattern of change in the environmental variables, the internal structure of populations, and the availability of adaptive pathways.

## 4. Materials and Methods

### 4.1. Virus Populations, Bacteria, and Standard Procedures for Infection

The plasmid pBRT7Qβ, containing the cDNA of bacteriophage Qβ cloned in the plasmid pBR322 [46,47], was used to transform *E. coli* DH5-α, which can support the expression of the virus genes and the assembly of virus particles, but cannot be infected by the virus because it lacks the F pilus. The supernatant of an overnight culture obtained from a transformed colony was used to infect *E. coli* Hfr (Hayes) in semisolid agar at a multiplicity of infection (moi) that allowed for the generation of well-separated lytic plaques. Under these conditions, there is a high probability that each lytic plaque results from the replication of a single virus for a limited number of generations, and, thus, they can be considered biological clones. The virus progeny contained in a randomly chosen lytic plaque was the origin of all the populations used in this study.

Standard infections in liquid medium were carried out in NB medium (8 g/L Nutrient Broth from Merck and 5 g/L NaCl), using fresh log-phase *E. coli* Hfr cultures with an OD_550_ between 0.6 and 0.8. After 2 h of incubation at 37 °C with good aeration (250 rpm), cultures were treated with 1/20 volume of chloroform for 15 min at 37 °C with shaking (300 rpm). Virus supernatants were harvested upon centrifugation at 13,000× *g*. Virus titers were determined by a plaque assay that was carried out by mixing 300 μL of exponential phase bacteria with 100 μL of the phage suspension in 3.5 mL of melted top agar, which was poured onto Petri dishes containing a bottom agar layer. The phage concentration was adjusted to obtain a number of lytic plaques between 20 and 40. Titers were expressed as the number of plaque forming units (pfu) per mL of the phage suspension in the case of virus supernatants.

The biological clone isolated upon expression of pBRT7Qβ was used to infect an *E. coli* Hfr culture as described above, using an moi of 0.1 pfu/cell in a volume of 1 mL (containing ~10^8^ bacteria). After 2 h of incubation at 37 °C, the virus supernatant was collected, and a fraction of it was used to infect a fresh *E. coli* culture, keeping the moi around 0.1 pfu/cell. The process was repeated for a total of 25 serial transfers. Populations obtained at transfer number 2 (Qβ-t2) or 25 (Qβ-t25) were propagated for 60 additional transfers but, instead of at 37 °C, the replication temperature was increased according to the different patterns shown in Figure 1. When the virus yield was too low to make infections at an moi of 0.1 pfu/cell, these were performed using the virus contained in 100 μL of the previous transfer. Evolutionary lineages were carried out in triplicate and corresponded to Qβ-t25 propagated through three patterns of change: sudden (lineages S-t25.1, S-t25.2, and S-t25.3), two steps (lineages TS-t25.1, TS-t25.2, and TS-t25.3), and gradual (lineages G-t25.1, G-t25.2, and G-t25.3); and to Qβ-t2 propagated only through the sudden pattern (lineages S-t2.1, S-t2.2, and S-t2.3).

### 4.2. Fitness Determinations

Duplicate liquid cultures containing 10^8^ bacteria growing in exponential phase were inoculated with 10^4^ pfu in a final volume of 1 mL. After two hours of incubation at 43 °C, the virus supernatants were collected as described above and titrated to estimate the virus yield, which was used as a surrogate of fitness. Previous assays showed that bacteriophage Qβ grew exponentially under these conditions. Absolute fitness was expressed as the number of doublings per two hours, and was calculated as log_2_[(*N_f_*–*N_0_*)/*N_0_*], where *N_0_* is the initial input of virus and *N_f_* is the number of progeny pfu. Relative fitness values were obtained by dividing the absolute values by the one of the corresponding ancestors (Qβ-t2 or Qβ-t25) determined in the same assay.

### 4.3. Determination of the Consensus Sequences of Virus Populations

RNA was extracted by treatment of the virus supernatants with 1/10 vol. 10% SDS, 1/10 vol. 50% β-mercaptoethanol, and 1 μL of RNasine for 20 min at room temperature with low shaking. After 5 min in ice, the sample was extracted with cold-water-saturated phenol and the upper phase was precipitated with ethanol following standard procedures. RNA samples were resuspended in RNase-free water and stored at −80 °C. RNAs were used for cDNA synthesis with the avian myeloblastosis virus reverse transcriptase (Promega), followed by PCR amplification using Expand high-fidelity DNA polymerase (Roche). The oligonucleotide primers used for PCR have been described previously [67,68]. PCR products were column-purified (Qiagen) and subjected to standard Sanger sequencing using Big Dye Chemistry with an automated sequencer (Applied Biosystems; Perkin Elmer). Sequences were assembled with SeqMan Pro (DNASTAR Lasergene 12 Core Suite) and aligned to the sequence of the cDNA of bacteriophage Qβ cloned in the plasmid pBR322 using the program Clustal X2.1 (Multiple Alignment Mode and default parameters). Mutations relative to this sequence were identified using BioEdit Sequence Alignment Editor. To make a more thorough analysis, the mutations, either fixed or polymorphic, that were detected in each evolutionary lineage were visually inspected in the chromatograms of the other lineages to evaluate their possible presence as polymorphisms not recognized by the sequence analysis programs.

## Figures and Tables

**Table 1:** Quantitative parameters of the fitness dynamics at 43 °C of the Qβ evolutionary lineages propagated through different patterns of temperature increase.

Evolutionary Lineage	Transfer Number (Relative Fitness = 2) ^1^	Relative Fitness at Transfer 60	Fitness Difference (First 10 Transfers at 43 °C)
S-t2.1	16.3	3.3	0.6
S-t2.2	16.9	3.0	0.3
S-t2.3	21.5	3.1	0.9
S-t2 (Mean ± SD)	18.2 ± 2.8	3.1 ± 0.1	0.6 ± 0.3
S-t25.1	8.1	2.7	1.2
S-t25.2	6.8	3.0	1.5
S-t25.3	7.0	3.1	1.4
S-t25 (Mean ± SD)	7.3 ± 0.7	2.9 ± 0.2	1.4 ± 0.1
TS-t25.1	30.3	2.4	0.9
TS-t25.2	28.5	2.6	0.7
TS-t25.3	27.8	2.7	0.8
TS-t25 (Mean ± SD)	28.9 ± 1.3	2.6 ± 0.1	0.8 ± 0.1
G-t25.1	43.7	2.5	-0.1
G-t25.2	45.5	2.3	0.02
G-t25.3	45.0	2.5	0.1
G-t25 (Mean ± SD)	44.7 ± 0.9	2.5 ± 0.1	0.01 ± 0.1

^1^ The transfer number at which relative fitness reached a value of 2 was determined through a linear interpolation of the data shown in Figure 3.

**Table 2:** Mutations present at transfer number 60 in the sets of lineages propagated through different patterns of temperature increase.

Mutation ^1^	Gene ^2^	Number of Lineages with the Mutation Per Treatment ^3^			
		S-t2	S-t25	TS-t25	G-t25
G502C (G147R)	A2				0/1
C714U (S)	A2	0/1			
A1088G (D342G)	A2	2/1	3/0	0/2	0/1
C1228U (L389F)	A2	0/1	1/0		
U1295(C/G) (F411S/C)	A2	1/0	2/0	1/2	1/1
G1312A (V417I)	A2	2/0	3/0	3/0	3/0
G1371A (G9S)	C	3/0	3/0	0/3	0/2
U1544C (S)	C		1/0		
G1607U (Q87H)	C		1/0		
C1649U (S)	C		2/0		
A1778G (S)	A1			1/1	1/0
C1806U (P154S)	A1			3/0	2/1
C1807U (P154L)	A1	1/0			
U1810C (I155T)	A1	1/0			
G1820A (S)	A1	1/0			
A1930G (Q195R)	A1				0/1
U2006G (S220R)	A1	0/2	0/1		
U2016C (F224L)	A1	1/0	2/1		
A2187C (S281R)	A1	0/3	0/1	3/0	2/1
C2201U (S)	A1				1/0
A2222C (S)	A1	1/0	3/0		
G2223A (V293I)	A1			1/1	0/1
C2384U (S)	R			0/2	
C2452U (A33V)	R	1/0			
U2776C (V141A)	R	2/0	1/1	3/0	3/0
G3002A (S)	R		1/0		
U3011C (S)	R		1/0		
U3311G (I320M)	R	1/0	3/0		
U3402C (S350P)	R	1/0			
U3447C (C365R)	R	0/1			
G3521U (E389D)	R		1/0		
C3545U (S)	R	0/1		3/0	2/1
G3896A (S)	R		0/1		
C3903U (L517F)	R	0/1	2/1		
G3945A (G531S)	R	2/0	3/0	3/0	1/2

^1^ The change of amino acid is shown in brackets. S indicates synonymous mutations. Position 1295 could mutate to C or G. ^2^ Location of the mutation in the different genes of the Qβ genome. C means coat and R replicase. ^3^ The first number of each pair indicates the number of lineages in a given set in which the mutation is fixed. The second number refers to the number of lineages in which the same mutation is present as a polymorphism. Empty cells indicate the absence of the mutation in that particular set of lineages.

**Table 3:** First substitutions fixed in the evolutionary lineages of bacteriophage Qβ and the number of the transfer at which they reached fixation.

Lineage	First Fixed Mutations	Number of Transfer
S-t2.1	A1088G, G1371A, C2452U, U2776C	30
S-t2.2	A1088G, G1371A, A2222C, U3311G	20
S-t2.3	G1312A, U3402C	10
S-t25.1	A1088G, C1649U, U2016C, A2222C, U2460C, U3311G, G3945A	10
S-t25.2	A1088G, A2222C, U3311G	10
S-t25.3	A1088G, C1649U, A2222C, U3311G	10
TS-t25.1	G1312A	20
TS-t25.2	G1312A, A1930G	20
TS-t25.3	G1312A	30
G-t25.1	G1312A	20
G-t25.2	A1930G	20
G-t25.3	G1312A	20

## Supplementary Materials

The following are available online at https://www.mdpi.com/2076-0817/8/2/80/s1, **Figure S1**: Mutations present in the consensus sequences determined every 10 transfers for the Qβ evolutionary lineages propagated through different patterns of temperature increase. The three squares below each mutation in each of the six rows shown in the figure correspond to the three replicate lineages at transfer number 10 (T10), 20 (T20), 30 (T30), 40 (T40), 50 (T50), and 60 (T60). A white square means the absence of the mutation. In colored squares, the intensity of the color indicates the relative amount of the mutated nucleotide according to the code shown in the figure. **Figure S2:** Dynamics of the accumulation of mutations in the consensus sequences of the Qβ evolutionary lineages. In each treatment, the blue line represents the evolutionary lineage 1, the orange line the lineage 2, and the grey line the lineage 3. The number of mutations includes both fixed and polymorphic mutations.
