# Supplementary material for: Evolutionary Dynamics in the RNA Bacteriophage Qβ Depends on the Pattern of Change in Selective Pressures
Source: Pathogens. 2019 Jun 18;8(2):80. doi: 10.3390/pathogens8020080 (PMC6631425; doi:10.3390/pathogens8020080)
Supplement: Supplementary file 1 [file pathogens-08-00080-s001.zip › Supplementary files/supplementary figure S1.pdf]

# Supplementary Figure S1

## Lineages S-t2

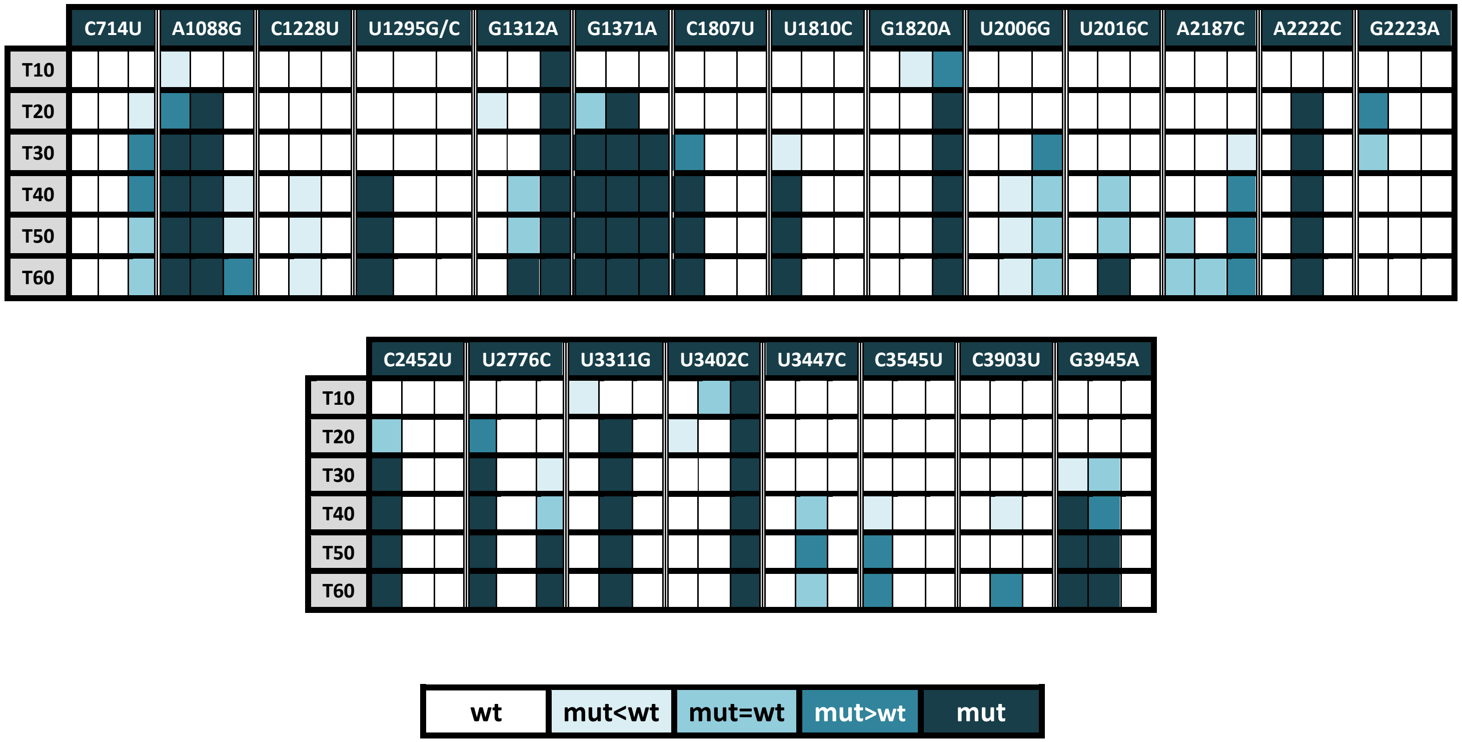

# Lineages S-t25

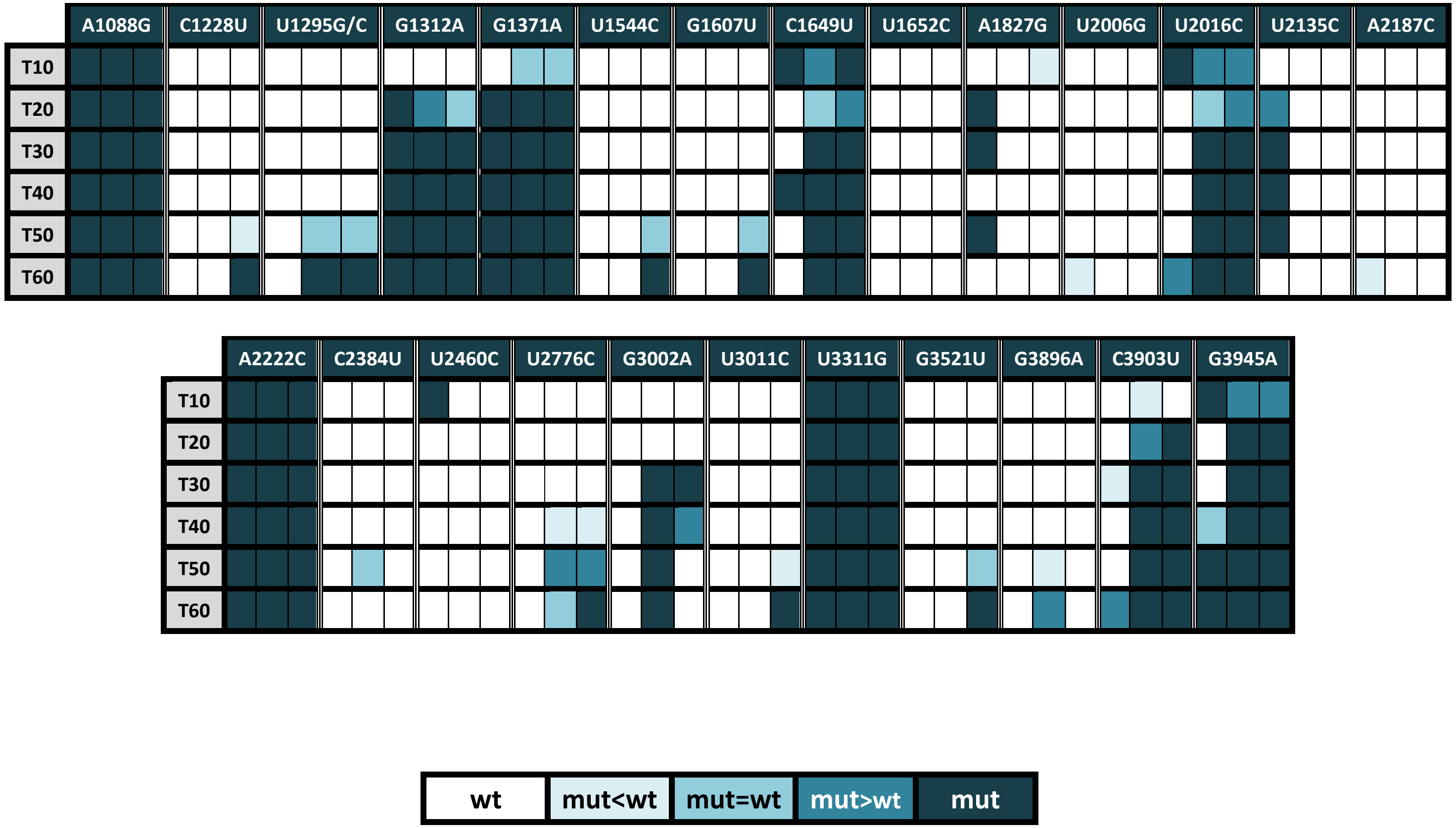

## Lineages TS-t25

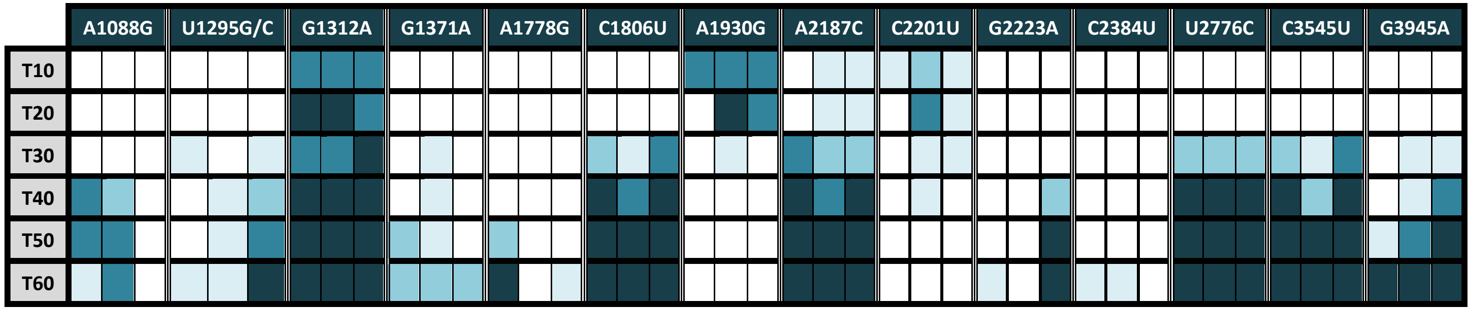


## Lineages G-t25

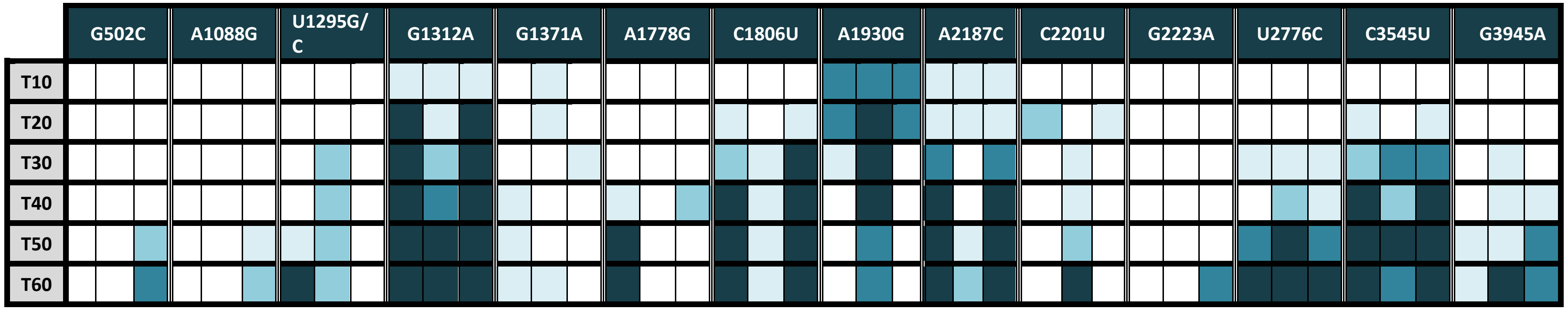


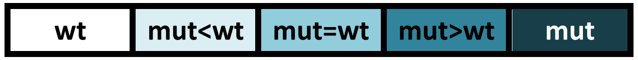


**Mutations present in the consensus sequences determined each 10 transfers for the Qβ evolutionary lineages propagated through different patterns of temperature increase.** The three squares below each mutation in each of the six rows shown in the figure correspond to the three replicate lineages at transfer number 10 (T10), 20 (T20), 30 (T30), 40 (T40), 50 (T50), and 60 (T60). A white square means the absence of the mutation. In colored squares, the intensity of the color indicates the relative amount of the mutated nucleotide, according to the code shown in the figure.
