# Supplementary material for: Evolutionary Dynamics in the RNA Bacteriophage Qβ Depends on the Pattern of Change in Selective Pressures
Source: Pathogens. 2019 Jun 18;8(2):80. doi: 10.3390/pathogens8020080 (PMC6631425; doi:10.3390/pathogens8020080)
Supplement: Supplementary file 1 [file pathogens-08-00080-s001.zip › Supplementary files/Supplementary Figure S2.pdf]

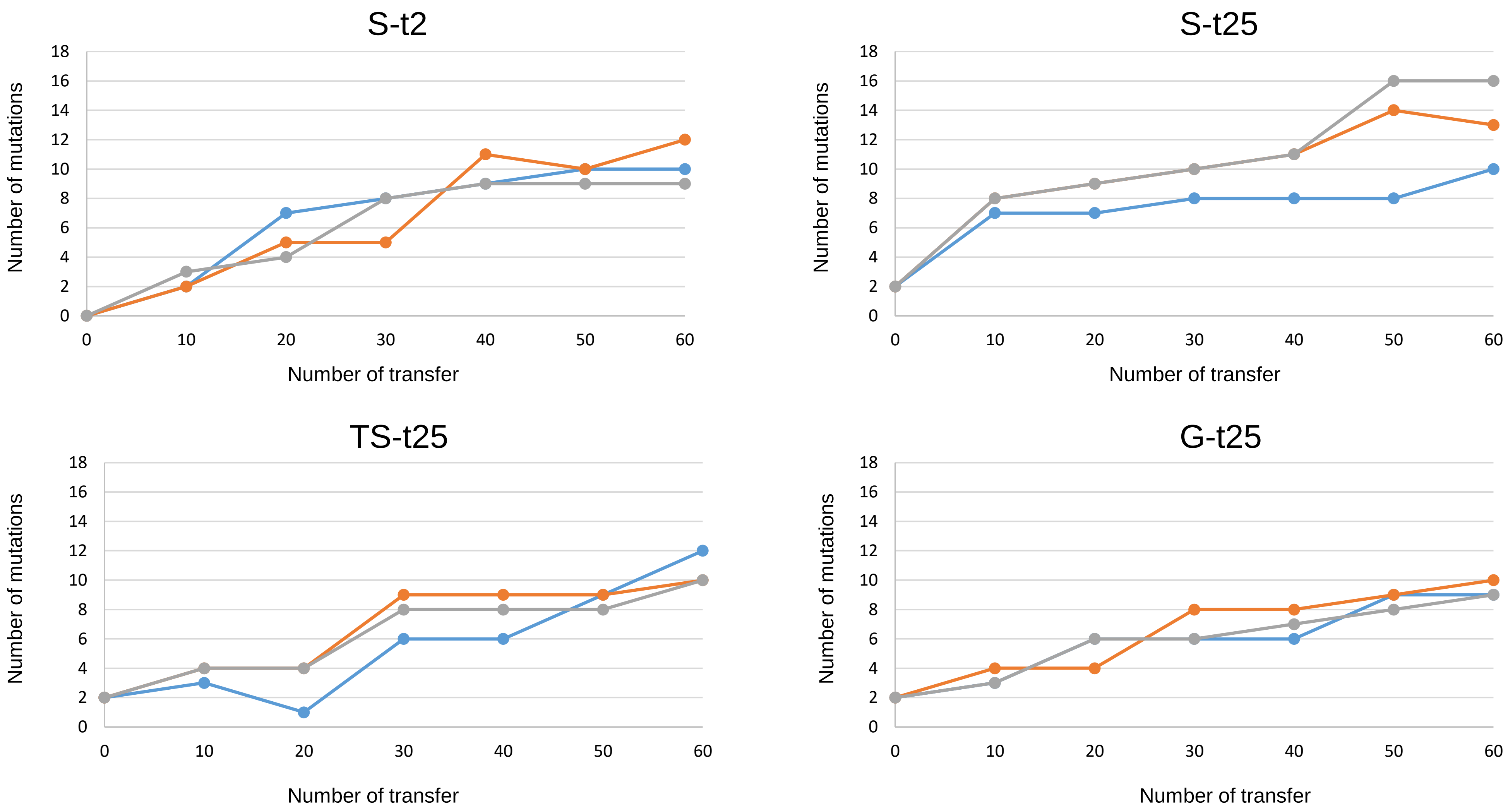

Supplementary Figure S2. Dynamics of the accumulation of mutations in the consensus sequences of the Qβ evolutionary lineages. In each treatment, the blue line represents the evolutionary lineage 1, the orange line the lineage 2, and the grey line the lineage 3. The number of mutations includes both fixed and polymorphic.
